# Supplementary material for: Early Humoral Response Correlates with Disease Severity and Outcomes in COVID-19 Patients
Source: Viruses. 2020 Dec 4;12(12):1390. doi: 10.3390/v12121390 (PMC7761967; doi:10.3390/v12121390)
Supplement: Supplementary file 1 [file viruses-12-01390-s001.pdf]

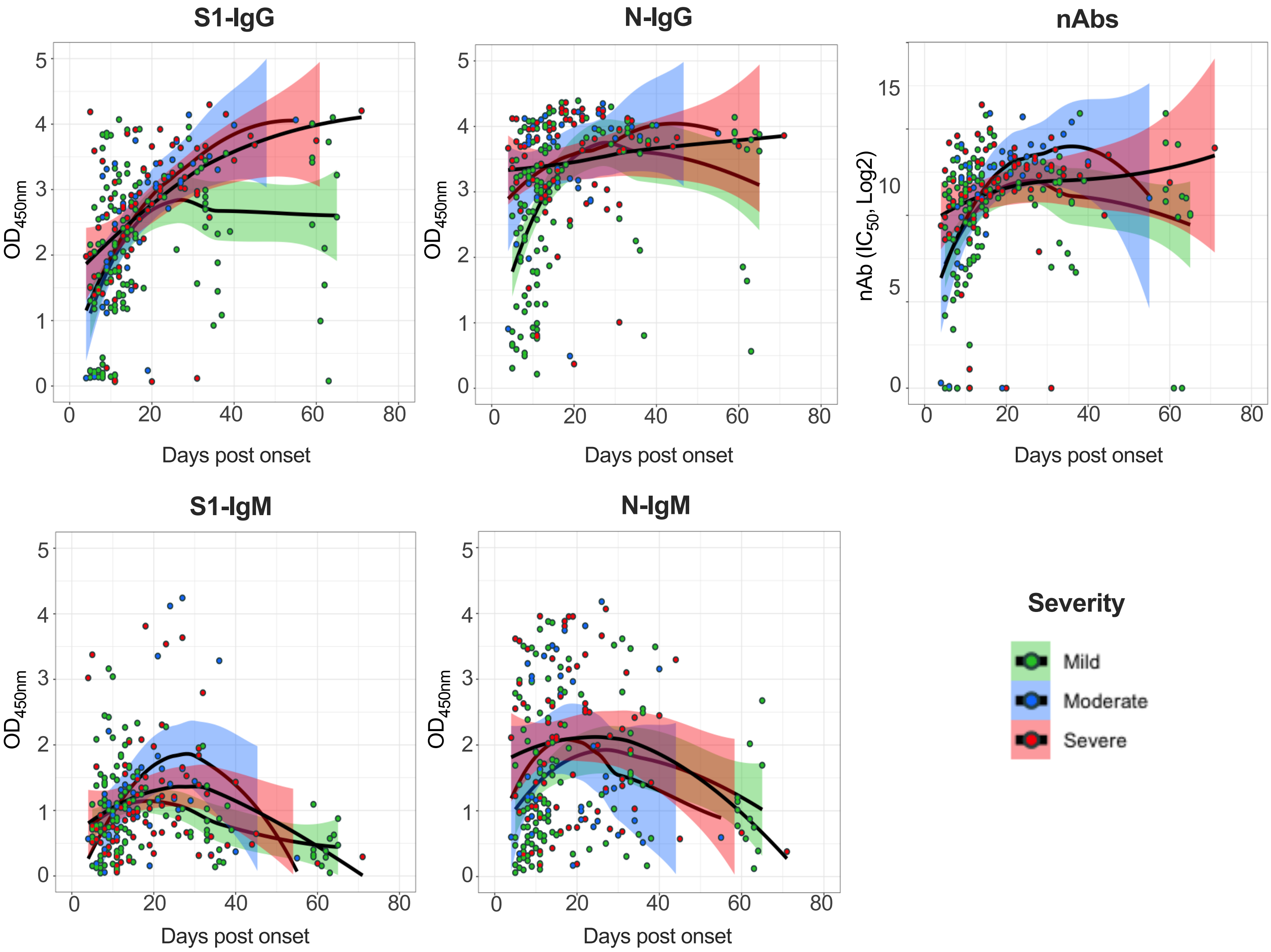

Supplementary Figure S1. Kinetics of SARS-CoV-2 specific antibodies over time based on disease severity. OD values of S1- and N-specific IgG and IgM (S1-IgG, S1-IgM, N-IgG and N-IgM) and neutralizing titers (IC_{50}) are plotted against the days post disease onset. The line and the ribbon show the mean expected from a LOESS regression model with 95% confidence interval for each disease severity category. The colored circles indicate disease severity. OD = optical density.

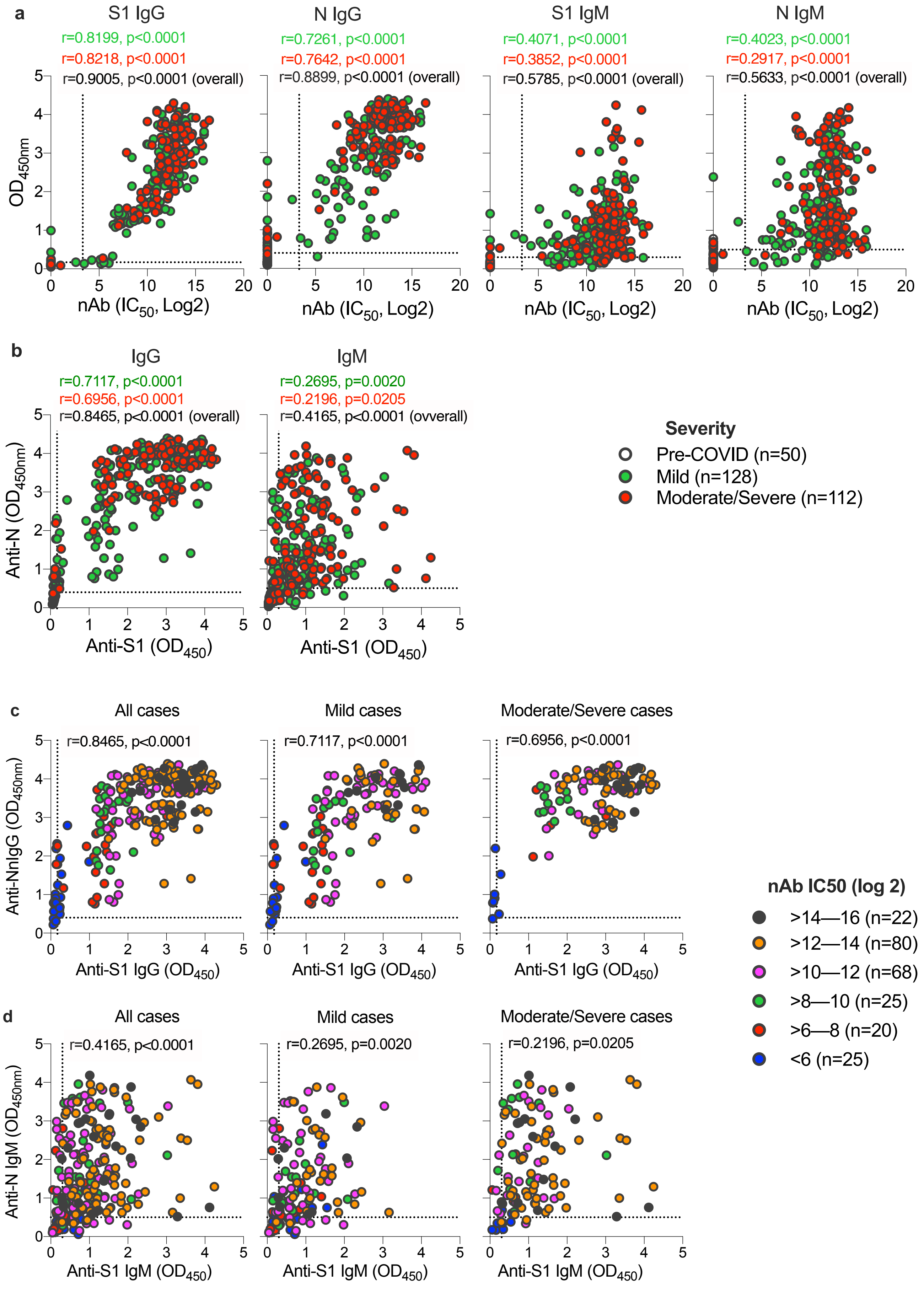

Supplementary Figure S 2. Relationship between S1 and N-specific IgG and IgM and neutralizing titer based on disease severity. (**a**) Pearson's correlation between S1-IgG, N-IgG, S1-IgM and N-IgM and neutralizing titer based on disease severity. Scatter plots were generated using OD values (y-axis) versus IC_{50} (x-axis) from each individual serum sample with colored circles indicate disease severity. (**b**) Pearson's correlation between IgG response against S1 and N proteins and IgM response against S1 and N proteins based on disease severity. Scatter plots were generated using anti-N OD values (y-axis) versus anti-S1 OD values (x-axis) from each individual serum sample with colored circles indicate disease severity. Pearson's correlation between (**c**) IgG response against S1 and N proteins and (**d**) IgM response against S1 and N proteins based on neutralizing titer. Colored circles indicate neutralizing titers (IC_{50}). The dotted lines represent the cut-off of each assay. OD = optical density.
